# Cognitive Behavior Therapy for Anxious and Depressed Youth: Improving Homework Adherence Through Mobile Technology

**DOI:** 10.2196/resprot.5841

**Published:** 2016-11-10

**Authors:** Pamela Wilansky, J Mikael Eklund, Tracy Milner, David Kreindler, Amy Cheung, Tim Kovacs, Shahin Shooshtari, Arlene Astell, Arto Ohinmaa, Joanna Henderson, John Strauss, Rosemary SL Mills

**Affiliations:** ^1^ Child, Youth and Family Services Centre for Addiction and Mental Health University of Toronto Toronto, ON Canada; ^2^ Department of Electrical, Computer and Software Engineering University of Ontario Institute of Technology Oshawa, ON Canada; ^3^ BrainFx, Inc. Pickering, ON Canada; ^4^ Division of Youth Psychiatry Sunnybrook Health Sciences Centre University of Toronto Toronto, ON Canada; ^5^ Centre for Mobile Computing in Mental Health Sunnybrook Health Sciences Centre University of Toronto Toronto, ON Canada; ^6^ Department of Computer Science Faculty of Engineering University of Bristol Bristol United Kingdom; ^7^ Department of Community Health Sciences Max Rady College of Medicine, Rady Faculty of Health Sciences University of Manitoba Winnipeg, MB Canada; ^8^ Ontario Shores Centre for Mental Health Sciences Whitby, ON Canada; ^9^ School of Health and Related Research University of Sheffield Sheffield United Kingdom; ^10^ School of Public Health University of Alberta Edmonton, AB Canada; ^11^ Shannon Centennial Informatics Lab Centre for Addiction and Mental Health University of Toronto Toronto, ON Canada

**Keywords:** mHealth, mobile app, youth, anxiety, depression, cognitive behavior therapy, homework

## Abstract

**Background:**

Anxiety and mood disorders are the most common mental illnesses, peaking during adolescence and affecting approximately 25% of Canadians aged 14-17 years. If not successfully treated at this age, they often persist into adulthood, exerting a great social and economic toll. Given the long-term impact, finding ways to increase the success and cost-effectiveness of mental health care is a pressing need. Cognitive behavior therapy (CBT) is an evidence-based treatment for mood and anxiety disorders throughout the lifespan. Mental health technologies can be used to make such treatments more successful by delivering them in a format that increases utilization. Young people embrace technologies, and many want to actively manage their mental health. Mobile software apps have the potential to improve youth adherence to CBT and, in turn, improve outcomes of treatment.

**Objective:**

The purpose of this project is to improve homework adherence in CBT for youth anxiety and/or depression. The objectives are to (1) design and optimize the usability of a mobile app for delivering the homework component of CBT for youth with anxiety and/or depression, (2) assess the app’s impact on homework completion, and (3) implement the app in CBT programs. We hypothesize that homework adherence will be greater in the app group than in the no-app group.

**Methods:**

Phase 1: exploratory interviews will be conducted with adolescents and therapists familiar with CBT to obtain views and perspectives on the requirements and features of a usable app and the challenges involved in implementation. The information obtained will guide the design of a prototype. The prototype will be optimized via think-aloud procedures involving an iterative process of evaluation, modification, and re-evaluation, culminating in a fully functional version of the prototype that is ready for optimization in a clinical context. Phase 2: a usability study will be conducted to optimize the prototype in the context of treatment at clinics that provide CBT treatment for youth with anxiety and/or depression. This phase will result in a usable app that is ready to be tested for its effectiveness in increasing homework adherence. Phase 3: a pragmatic clinical trial will be conducted at several clinics to evaluate the impact of the app on homework adherence. Participants in the app group are expected to show greater homework completion than those in the no-app group.

**Results:**

Phase 3 will be completed by September 2019.

**Conclusions:**

The app will be a unique adjunct to treatment for adolescents in CBT, focusing on both anxiety and depression, developed in partnership with end users at every stage from design to implementation, customizable for different cognitive profiles, and designed with depression symptom tracking measures for youth made interoperable with electronic medical records.

## Introduction

### Background

Mobile technology has the potential to make mental health treatments more effective and efficient in alleviating mental health problems [1-4]. In particular, youth anxiety and depressive disorders may benefit from the use of mobile technology to improve treatment. The incidence of these disorders peaks during adolescence [5-7], putting them among the top 5 causes of illness and disability in the world [8]. Their persistence into adulthood often takes a great toll on daily functioning in social, work, and family contexts, reducing health-related quality of life [9,10], and placing a heavy financial burden on society and health care services [11,12]. Given the long-term impact of these disorders on individuals, families, and society and their high economic burden, increasing the effectiveness of early treatment could have a significant impact. Cognitive behavior therapy (CBT) is the established efficacious treatment for anxiety and depressive disorders. It has been shown to be an effective treatment for anxiety and depression in children and youth across a wide range of ages [13-19] and using various modes of delivery (eg, individual and group) [20,21]. Notwithstanding CBT’s effectiveness, many individuals are not successfully treated and continue to have significant symptoms [22-24]. For example, in a systematic review of CBT for anxiety in young people, anxiety diagnoses were still present at the end of treatment in more than one-third of participants [23]. Increasing the success of CBT in treating adolescents would result in a lower rate of relapse. A promising target for increasing the success of CBT is the homework component.

### The Role of Homework in Cognitive Behavioral Therapy Success

The theory underlying CBT combines cognitive and behavior theories to suggest that negative thinking patterns and learned responses underlie emotional responses and behaviors [25,26]. Treatment aims at helping adolescents recognize the links between maladaptive thoughts, negative emotions, and maladaptive behaviors in order to replace them with more positive thinking and adaptive behaviors. The acquisition of new ways of thinking and new behaviors occurs through learning processes: cognitive learning, classical and operant conditioning, shaping, maintenance, and generalization. As in all learning, practice is essential. New concepts and skills introduced in the treatment session are practiced in problematic situations outside the session to promote experiential learning and generalization to new situations in daily life. For example, graduated exposure to feared situations is used to lessen anxiety. Behavioral activation (eg, engaging in pleasurable or mastery activities) is used to reduce depressed mood. Activities such as these comprise the homework assignments to be carried out between sessions, selected together with the therapist, in order to aid progress toward therapy goals [27]. Thus, given the centrality of experiential learning in CBT, homework is an essential component of treatment.

Typical therapy sessions last an hour a week and in large measure are devoted to planning and processing the efforts made outside the session [28]. At the end of the session, a homework assignment is introduced and explained, usually consisting of a practice exercise based on what was learned in the session, outlined on a worksheet with space to complete the task. For example, an adolescent might be asked to complete a thought record about an upcoming anxiety-provoking situation, such as a math test at school. The worksheet is to be completed and returned at the next session. The session begins with a review of the homework to reflect on the task as well as the difficulties that may have been encountered and what has been learned [29]. Treatment guidelines for administering homework are designed to support the conduct of homework: assignments should be meaningful, relevant to the central goals of therapy, relevant to the focus of the therapy session, agreeable to patient and therapist, appropriate to the patient’s sociocultural context, doable (concrete, specific, and appropriate to current skill level and level of functioning), have a clear rationale, include a backup plan that anticipates potential obstacles and how to handle them, be initially practiced in the session, and include written instructions [30]. These guidelines are designed to ensure that patients are prepared to continue practicing during the week in between therapy sessions.

### Support for Practice and Learning

Reflecting homework’s critical importance for the success of treatment, there is a direct association between homework completion and outcomes of CBT for anxiety or depression across a wide age range [28,31-34]. However, rates of homework completion are uniformly low across the age spectrum. Studies of adolescents in CBT for depression show that completion rates hover at approximately 50%, are highly variable, and tend to decline across sessions [28,35,36]. These findings may be partly attributable to insufficient support for practice and learning during the interval in between therapy sessions. Although the guidelines for administering homework are intended to ensure that practice occurs, much depends on the patient’s ability to continue practicing without the support that was provided during the treatment session. Whereas in-session learning occurs with the help of a therapist who provides encouragement, feedback, and problem-solving support, learning outside the session is a self-directed effort. The absence of the therapist’s support during the intervals between sessions may jeopardize the conduct of homework assignments.

Consistent with this interpretation, adolescents’ reflections on CBT homework suggest that they experience insufficient support for doing homework. In a CBT program for depression [37], adolescents regarded homework as important, but reported not always completing the assigned exercises because they did not feel motivated or found it too time-consuming. In another CBT program for depression, which consisted of a computerized program designed as a self-help computer game [38], adolescents gave various reasons for not completing homework, including lack of interest and not having a helpful resource. These findings suggest that homework as traditionally administered provides insufficient support for learning. Rates of homework completion may improve if greater support were provided. Adolescents especially are likely to benefit from enhanced support. Adolescence is a period of ongoing cognitive development involving growing understanding of abstract concepts relevant to CBT, and thus a time when support for learning is likely to be particularly important [39].

### The Role of Mobile Tools

Mobile tools have the potential to facilitate many of the treatment processes involved in CBT [2,40]. In particular, given the essential role of homework in CBT, they have been conceptualized as a means by which the therapy setting can reach beyond the clinic to the patient’s everyday environment [41]. A mobile homework app may support learning between sessions in numerous ways, including making homework materials accessible and easy to keep track of; aiding memory and understanding of the lesson learned during the treatment session; providing coaching and suggestions (eg, through a help function); promoting intrinsic motivation (eg, through goals and challenges, rewards, feedback on progress [42,43]); facilitating self-monitoring of symptoms and changes (eg, through visual displays); and enhancing homework review and troubleshooting (eg, by summarizing results for discussion). In summary, mobile delivery of homework may provide a means of supporting the continuation of learning during the intervals in between treatment sessions.

The purpose of the proposed research is to design and evaluate a mobile CBT homework app that provides a support system for young people in therapist-led treatment for anxiety and/or depression. We expect that the app will improve homework completion by supporting learning; that is, by facilitating access to assignments, memory and understanding of lessons, motivation to practice, self-monitoring, and review of homework results. Given the impact of cognitive skills on learning, an app is most likely to support learning if it is delivered in a manner consistent with the patient’s cognitive skills (eg, abstract reasoning, and executive functioning skills such as planning, implementing, and reflecting) and presented in a way that is meaningful. To this end, the proposed app will be designed to enable analytics for ongoing improvement and customization for different patterns of cognitive strengths and challenges compared with others (cognitive profiles) [44,45].

### Current Empirical Support for Cognitive Behavioral Therapy Homework Tools

There are very few existing evidence-based mobile mental health apps [46-48]. Only a handful of apps are relevant to CBT homework and all of these are in the early stages of development. Of those designed for adolescents, several focus specifically on self-monitoring: *Mobile Mood Diary* [49,50], *mobiletype* [51-55], and a daily pain diary [56,57]. Research on these tools suggests that they are useful and acceptable to adolescents. Adolescents complied with daily diaries and momentary sampling and seemed to prefer mobile versus paper methods for self-monitoring. Mood graphs appeared to facilitate discussion in the therapy session. An intervention to facilitate self-monitoring as well as skills practice for pain management was evaluated as usable and acceptable by adolescents and their parents, and preliminary evidence indicated that it had a beneficial effect on coping skills [57].

One mobile app provides full homework support for youth in CBT: Smartphone-enhanced Child Anxiety Treatment (SmartCAT [58]), a comprehensive system to support clinician-directed CBT treatment for anxiety. SmartCAT is designed to enhance the practice of CBT skills outside the clinic by reminding children to practice, motivating practice through rewards, enabling personalized support by the therapist, and facilitating patient-therapist interaction. The central feature of the app is a skills coach, which delivers ecological momentary interventions by cueing children to answer a series of questions about recent events and guiding them through a series of steps. A feasibility study conducted with 9 anxious youth between 9 and 14 years of age indicated good compliance with the skills coach (82.8% response to cueing). Participants rated the app as highly usable.

Several apps relevant to CBT homework have been developed for adults, all providing full homework support: *PE Coach* [59,60], *PsychAssist* [61], and a general therapy support system [62]. All 3 systems contain psychoeducation and homework assignments for each component of treatment. They also include forms for completing activities and a system for scheduling activities and sending reminders. With the exception of *PE Coach*, they are equipped with separate interfaces for patient and therapist and include features to facilitate the review and troubleshooting of homework. All have been positively evaluated as easy to use and helpful. While their clinical effectiveness has yet to be examined, there is preliminary evidence that the general support system [62] increases homework completion and reduces symptoms. How suitable the systems are or how easily they could be adapted for youth is unknown.

In summary, a small handful of CBT homework apps exist ranging in scope from specific to comprehensive and varying in their stage of development. User evaluations indicate that the apps are perceived as useful and acceptable. The findings provide tentative support for several features: ready access to each assignment along with material to aid memory and understanding of lessons and the purpose of the assignment; reminders to complete homework; a skills coach; a means of obtaining personalized support; and graphics depicting trends over time to facilitate homework review and reflection on progress. However, it remains unclear how usable and acceptable these designs are. With the exception of *Mobile Mood Diary,* user evaluations were conducted following the design stage, which risks constraining the evaluation and requiring significant redesign later on. A guiding principle of user-centered design is that end users be involved from the outset of the design stage in order to ensure good usability [63,64]. As well, with the exception of *PsychAssist*, user evaluations were conducted in a single round instead of iterative rounds involving modification of the design followed by evaluation of the new design. An iterative process of evaluation will ensure good usability by progressively eliminating usability problems until no further significant problems are identified.

We will address these methodological issues by including end users from the outset and conducting usability evaluations iteratively. We will also adhere to a design approach recommended for sensitive areas like mental health [65,66], in which design begins in a nonclinical context to ensure a safe design before evaluating usability in the context of treatment (see below). In addition, the design will include a data collection system that enables analytics for ongoing improvement and customization of the app for different cognitive profiles. As well, we will use new technology described below to support our app’s client self reports use with electronic medical records (EMRs). The expansion of EMR use in clinical care has underscored the dated method most clinicians use to collect patient self reports (ie, paper). Finally, we will move the app from research into practice through a collaborative process guided by integrated knowledge translation and implementation frameworks [67-69]. This involves collaboration among those who develop, deliver, and support the innovation guided by a plan that is monitored and evaluated at each step [68-70].

### Objectives

The purpose of the proposed research is to design and evaluate a mobile CBT homework app for adolescents to use as an adjunct to therapist-led treatment for anxiety and/or depression. The objectives are to (1) design and optimize the usability of a mobile app for delivering the homework component of CBT for youth with anxiety and/or depression, (2) assess the app’s impact on homework completion, and (3) implement the app in CBT programs.

## Methods

### Feasibility and Requirements Analysis

The growth of wireless communications supports the feasibility of using mobile technology in CBT. In 2014, wireless networks reached 99% of Canadians [71]. Smartphones are a common part of everyday life for growing numbers of Canadian adolescents. Rates of cellphone usage have been increasing across a wide range of groups. Cellphone users between the ages of 14 and 17 have led the way in recent years, as shown by a 75% increase in usage rates among youth between 2011 and 2012, and usage among adults is catching up [72,73]. US data indicate that youth, minorities, and those with low levels of income and education have higher-than-average rates of cellphone usage, suggesting that high usage is not confined to privileged groups, although access to service may be more tenuous among those with lower incomes [74,75]. Cellphone use among anxious and/or depressed adolescents may be even higher than that of other adolescents, given research showing that greater use of text messaging and talking via the Internet was associated with higher social anxiety [76].

The objective is to develop an app that can be used as an adjunct to therapist-directed treatment to support homework completion. The software app will be built for multiple operating system platforms that permit the transmission of data to and from a robust, secure, and reliable database server and a fairly small local app. This enables the design of a tool with rich graphics and interactive features without requiring a large memory capacity and data storage on the user’s device (smartphone, tablet, or computer). Data will be stored on the server instead of the device itself, allowing for the proposed analytics. There will be a small local app so that, when WiFi is not accessible, data can be stored on the app and uploaded later. Having a fairly small local app will make it easier to install the app, and provisions will be made to allow for a basic data plan to be sufficient for the end user. With a password-protected account, users will be recognized by the server and, upon logging in, the app will connect to the CBT data on the server. Each time users log in, they will be able to pick up where they left off. A separate portal for therapists will be included to facilitate the review of homework by providing access to homework results if adolescents give consent; other functions may be added, contingent on design input from end users. We will use an HTML5-based framework providing an interactive experience on the majority of mobile devices and platforms including iOS and Android.

The server will consist of a database and the necessary server software to enable secure connectivity by the mobile apps and users. The database will store the assignment and the patients’ responses/entries along with timestamp information for analytical use for both optimization of the system and assessment of the app’s effectiveness.

### Interoperability With Electronic Medical Records

We will build public domain depression symptom tracking measures for patients’ treatment response that will be made interoperable with EMRs via an HL7-based health information technology (IT) platform [77], which uses open standards for health data, authorization, and user interface integration for full HL7 interoperability with a variety of EMRs from different vendors. Harvard University and Boston Children’s Substitutable Medical Applications and Reusable Technology (SMART) apps [78] is a health IT platform for creation of third party apps using open source application programming interface (API) with well-defined data models that predictably presents specific patient-level data. Multiple apps have been created using SMART, and recently the new HL7 Fast Healthcare Interoperability Resources (FHIR) specification has been added to SMART. FHIR uses a RESTful API for queries, in addition to standard data models and Web formats such as JSON and XML. FHIR can be used as a standalone interoperability standard, or together with existing widely used standards, such as HL7 v2 and v3.

How will SMART on FHIR mitigate interoperability gaps? Historically it has been difficult to get information from patients into EMRs. Various media were used (eg, external hard disk drives and USB drives). Without a usable interface, human delivery (aka, the sneakernet) was a main way to enable patient data to be entered or imported into an EMR. SMART on FHIR solves the sneakernet problem by using (1) an international health information standard, HL7, which has been embraced by all major EMR vendors, and (2) flexible, modular Web-based APIs (apps) that support external reading from or writing to the EMR. For these reasons, the recent arrival and early success of SMART on FHIR technology has been met with great enthusiasm by the medical informatics community and large EMR vendors.

### Overview of Approach

The essential content of the app will consist of practice exercises drawn from the manual of an empirically supported 12-week CBT program for younger adolescents [79-80]. The user interface and other features of the app will be designed and evaluated in 3 phases following a user-centered design approach. An easy-to-use software interface is essential to the effectiveness of a tool and it should be as good as it can be before the tool is tested for its effectiveness in improving treatment processes and treatment outcomes. Therefore, we will optimize usability before evaluating clinical effectiveness. The process is user-centered from the outset involving end users (youth, therapists) participating in multiple iterative rounds of design, testing, redesign, and retesting until the interface is deemed easy to use, acceptable, and ready for implementation [64]. In the area of mental health, ethical guidelines emphasizing the protection of patients against harm [65,66] suggest that, to ensure a safe design, the process should begin outside the context of treatment with end users who are similar to the target patient end users but are free of diagnosed mental health problems. Once the design is deemed safe, further rounds of testing and refinement are conducted with target end users who are diagnosed and in treatment. Finally, clinical testing is done to evaluate the impact of the tool on treatment processes and outcomes.

Following these guidelines, the development of the app will involve 3 phases: an initial stage of prototype design and usability evaluation conducted outside the context of treatment to ensure a safe design (Phase 1) followed by usability optimization conducted with patients in treatment (Phase 2), and finally, an effectiveness study to assess the effects of the app on homework completion and explore its impact on treatment outcomes and treatment cost-effectiveness in a pragmatic clinical trial (Phase 3).

### Implementation Plan

To ensure successful implementation of the app across Canada, the project team includes an experienced health technology partner and multiple stakeholder representatives (end users, researchers, treatment providers, health system decision-makers) who will work collaboratively to review results and make design decisions at every step, assist in driving implementation during and post project, and evaluate the quality of the implementation process. Our technology partner, BrainFx, Inc., will lead the app’s commercialization including ongoing collaboration with the implementation team for maintenance, updates, and enhancements that will continue to keep the app relevant and responsive beyond the project. In addition to their experience in developing and commercializing a digital clinical assessment tool (BrainFx 360), they provide expertise in neurofunctional assessment and in advanced analytics to support ongoing improvement and customization of the app for different cognitive profiles. Treatment providers represent 5 test sites that were chosen based upon their varied geographic location (eg, rural vs urban), type of setting (eg, community vs psychiatric hospital vs general hospital with psychiatric division), specialization (eg, generalist clinics vs mood and anxiety specialization; psychiatric hospital vs youth-focused hospital), and interprofessional staffing (eg, psychiatrist, psychologists, nurses, social workers). These variations will facilitate the identification of implementation barriers and generalization of the results from the present study to other locations. The test sites have also been chosen for their commitment and ability to embed and sustain the app in their current practice based on having clinicians who practice CBT with youth who are anxious and/or depressed. They include the Centre for Addiction and Mental Health (Toronto, ON), Canadian Mental Health Association (York Region & South Simcoe, ON), SickKids (Toronto, ON), Markham Stouffville Hospital (Markham, ON), and Sunnybrook Health Sciences Centre (Toronto, ON).

### Phase 1: Prototype Design and Usability Optimization in a Nontreatment Context

The purpose of Phase 1 is to design and develop a fully functional (programmed) prototype with input from adolescents and therapists who are familiar with CBT, and thus able to contribute to the design of a CBT homework app.

#### Exploratory Interviews

First, exploratory interviews will be conducted individually or in small focus groups of adolescents and of therapists to obtain participants’ views and perspectives on the requirements and features of a usable design and on issues pertaining to implementation. Participants will receive a CAD$30 gift card as an honorarium.

##### Sample

We will recruit (1) up to 10 adolescents between 12 and 18 years of age who can read and speak English, do not have a profound learning disability that could interfere with engagement in CBT, and have had some experience of CBT for anxiety and/or depressive disorders (have previously been or currently are in CBT), and (2) up to 10 CBT therapist (eg, psychologist, social worker, nurse, or occupational therapist) who have led at least 2 CBT groups for anxious and/or depressed adolescents and/or provided individual CBT to at least 5 anxious and/or depressed adolescents.

##### Procedure

Interviews with adolescents and therapists will be video and audiotaped. Following a warm-up discussion about their use of mobile apps in general (what apps are appealing, how they choose apps), adolescents and therapists will be asked about their experience with CBT homework, using a list of homework activities as a reference: what challenges were involved in doing/administering homework, what they liked and what they disliked about it, any suggestions for improving it, and whether a mobile app would be a helpful tool. To guide design decisions, they will also be asked to provide input on potential design features, such as reminders to do homework (what form, how frequent), rewards for doing exercises (what kind), tips to get unstuck, a way to get feedback and help (what kind, from app or therapist), a way to display homework at the next session, and whether therapists would like to have a separate portal (serving what functions). Similarly, they will be asked for design advice to make the app appealing to use for youth and therapists (eg, colors, navigation tools). Finally, they will be asked about the contexts in which a CBT homework app would likely be used (where, when, and how), and any concerns regarding implementation (eg, access to WiFi, privacy and security, availability of support, negative effects).

##### Data Analysis

The video and audiotapes will be transcribed and analyzed using conventional content analysis [81] to group statements into themes, issues, and suggestions in order to reveal insights, ideas, or concerns. We will review the results to settle on the design of an initial prototype. This design will then be programmed and made compatible with major operating systems (eg, Apple iOS, Android, Windows Phone) and accessible from smartphones.

#### Think Aloud Study

Next, a think aloud study will be conducted to evaluate and optimize the usability of the initial prototype through an iterative process of evaluation, modification, and re-evaluation.

##### Sample

Optimization will be conducted with a sample of up to 10 youth between 12 and 18 years of age who can read and speak English, do not have a profound learning disability, and have had some experience of CBT for anxiety and/or depressive disorders, and up to 10 CBT therapists. Participants in the exploratory interviews may be included in the think aloud sample. The sample size is based on evidence regarding the number of evaluators typically required to reach saturation (ie, to detect most usability problems, ~5) [82,83] and allows for more evaluation cycles than may be required. The sample will be distributed across iterative cycles of evaluation and improvement, each involving up to 5 evaluators whose feedback is used to modify and reprogram the prototype for the next cycle. Cycles continue as long as new problems are identified. Two cycles are often sufficient [84,85].

##### Procedure

The think aloud method [65] involves verbalizing thoughts while performing a task. It is effective for usability testing because it helps identify which interface features users find intuitive and easy to use and which require improvement and further evaluation [65]. Participants will be scheduled for individual video and audiotaped sessions with an interviewer in a quiet setting that facilitates thinking aloud. Adolescents and therapists will both be given tasks to complete with the user interface for patients (eg, finding specific pieces of information, navigating to a specific part of the app, doing an exercise). They will be instructed to verbalize their thoughts continuously as they work through the tasks, while the interviewer makes field notes of problems observed. If the prototype design includes a separate therapist interface, therapists will complete several additional tasks using that interface.

A heuristic evaluation by usability specialists will also be conducted. Up to 5 mobile app developers will be asked to inspect the user interface and independently rate the extent to which it meets established usability principles for software systems [86] (eg, error prevention, recognition and recovery from errors, aesthetic and minimalist design, help and documentation).

##### Data Analysis

Think aloud transcripts and interviewer field notes will be analyzed by performing a content analysis [87] to reveal issues with usability, such as unclear instructions, unintuitive icons, and difficult navigation sequences. Following each iterative cycle, we will review the results and modify the prototype for the next cycle. The process will culminate in a usable design that is ready for further evaluation and optimization in a clinical context.

### Phase 2: Usability Optimization in the Context of Clinical Treatment

The purpose of Phase 2 is to evaluate and improve the usability of the prototype in the context of a 12-week course of CBT treatment. Associations between cognitive profiles and user experience will be explored to better understand how to customize the app for different cognitive profiles. This phase will result in a usable app that is ready to be tested for its effectiveness.

#### Sample

An independent sample will be recruited consisting of 20 youth in CBT and their therapist **s**, distributed approximately equally across 5 clinics that provide CBT treatment for youth with anxiety and/or depression. Participants will receive a CAD$30 gift card as an honorarium. Youth will be recruited from among 12- to 18-year olds with a primary diagnosis of anxiety (general anxiety disorder, separation anxiety, social anxiety, panic disorder) and/or depression (major depressive disorder, dysthymic disorder). Informed consent will be obtained from youth, their primary caregiver, and their therapist. Youth receiving medication will be included if they were on a stable dose for approximately 6 weeks prior to and throughout participation. Those with a primary diagnosis of obsessive-compulsive disorder or post-traumatic stress disorder, and those with comorbid psychosis or substance dependence will be excluded because they require different CBT strategies from those that are the focus of this research. Those who are behind by 2 or more school grades or have a profound learning disability will be excluded, given the cognitive and verbal nature of CBT. Substance use and mild learning difficulties will not be exclusion criteria. Based on intake rates of approximately 1 per week at each clinic, we expect to recruit 5 youth per week. Interest in trying the app is expected to be high. Two evaluation cycles, each with up to 10 youth and their therapists, should be sufficient to reach saturation [82], but the sample size allows for an additional cycle if needed. Youth will be given the choice of receiving a smartphone to use or using their own. They will be compensated for the cost of a basic service plan (voice, text, Internet) for 12 weeks to use the app.

#### Sample Characteristics

To describe the sample, confirm diagnoses, and explore how individual attributes may affect the user experience, youth will be assessed using the following measures administered to them and their primary caregiver/guardian by a clinical psychology graduate student research assistant (RA) trained in the administration of the measures: the Achenbach Scales [88] to assess general symptomatology, completed by youth and caregiver; the Multidimensional Anxiety Scale for Children 2^nd^ ed. (MASC-2 [89]) completed by youth and caregiver; the Child Depression Inventory 2^nd^ ed. (CDI-2 [90]) completed by the youth; the Anxiety Disorders Interview Schedule (ADIS [91]) conducted with youth and caregiver; and selected tests from the BrainFx 360 digital clinical assessment of neurofunction [92] (performance assessment by a trained administrator focusing on complex cognitive skills: divided attention, delayed memory for auditory and visual input, mental flexibility, abstract reasoning, executive functioning skills in the areas of planning, organizing, implementing, and reviewing/reflecting). The Achenbach Scales, MASC-2, and CDI-2 will be administered in a secure Web-based format. The ADIS is a semistructured interview that will be administered either over the phone or in person according to preference. Both methods have been shown to be reliable [93]. The ADIS is the optimal research interview to assess anxiety disorders, but also includes all Diagnostic and Statistical Manual of Mental Disorders criteria for depression. In addition, information will be obtained about demographic characteristics (youth age, gender, and grade; parental education, occupation, and family income) and comfort with technology (experience with computers, smartphones, tablets, and the Internet–where used, frequency of use, comfort level).

#### Procedure

Prior to beginning CBT treatment, the RA will meet individually with youth to explain the purpose of the evaluation, describe the procedure for obtaining their feedback on the usability of the app, and administer the pretreatment assessment. Before the first therapy session, the site coordinator will meet with each youth to demonstrate how to use the app and provide contact information in the event of technical problems, and with therapists to train them on the app and how to use it to introduce and review homework. Therapists will meet with the coordinator periodically to discuss experiences and any technical issues.

The usability evaluation by youth will be done via a short weekly interview conducted upon arrival at the therapy session. The interview will focus on the app’s usability for the assignment given at the end of the previous session. We will assess the efficiency of the app, via ratings of the app’s learnability (how easy to figure out and remember what to do), ease of use (the time and number of steps it takes to operate, need for technical support), and error tolerance (how free of error messages, app’s ability to prevent/recover from use errors). We will also assess satisfaction with the app, via subjective ratings of its learnability (access to the assignment, memory and understanding of the lesson, motivation to practice, coaching and suggestions, self-monitoring of symptoms and changes), usability (how easy it was to use), and likeability (of specific design features). In addition, open-ended questions will be asked about: (1) where, when, and how the app was used (to understand context of use), (2) problems using the app (to identify usability problems), and (3) how the app supported homework completion (to understand the usefulness of the app). The final evaluation interview will include additional items focusing on overall experiences and impressions of the app, ratings of the app’s overall efficiency (learnability, ease of use, error tolerance), and overall satisfaction with the app (usefulness, usability, likeability). Open-ended questions will be asked about features most and least liked, facilitators and barriers to use, any negative experiences associated with the app (eg, intrusion on privacy or fear of such an intrusion), how much the app supported learning (ratings of support for access to assignments, memory and understanding of lessons, motivation, coaching and suggestions, self-monitoring, review of homework), and any suggestions for improvement.

The usability evaluation by therapists will be done via an interview following the final session, through open-ended questions about the usefulness of the app for reviewing and troubleshooting homework, whether there were any negative effects of using the app, and any suggestions for improvement.

A heuristic evaluation by usability specialists will also be conducted, as in Phase 1. Up to 5 mobile app developers will be asked to inspect the user interface and independently rate the extent to which it meets established usability principles for software systems [86] (eg, error prevention, recognition and recovery from errors, aesthetic and minimalist design, help and documentation).

#### Data Analysis

Content analysis will be performed on responses to open-ended questions to reveal contexts of use, usability issues, features most and least liked, facilitators and barriers, any anticipated negative effects, usefulness of the app for homework completion, and suggestions for improvement. Responses will be summarized in narrative form and frequencies of each category will be calculated. Descriptive statistics will be performed on the Likert-type ratings to measure central tendency (mode) and variability (frequencies). The cognitive skills assessment will yield clustering profiles outlining areas of strength and challenge as compared with others, permitting an exploratory qualitative analysis of associations between cognitive profiles and the usability evaluation data. The results of the study will be used to determine whether the app is usable and safe to use in the context of treatment, and thus is ready for a clinical trial of its effectiveness and acceptability in a treatment context, and to further improve the app before proceeding.

### Phase 3: Evaluation of Effectiveness

The purpose of Phase 3 is to evaluate the impact of the app on homework completion. We hypothesize that participants in the app group will show greater homework adherence than those in the no-app group. To test the hypothesis, a pragmatic clinical trial will be conducted at the same 5 clinic sites as in Phase 2. CBT is well supported by randomized controlled trials of its efficacy when delivered in different ways, including computerized formats as described above. Therefore, the purpose of this phase is not to determine CBT efficacy, but rather to provide evidence that homework (ie, the practice of CBT skills and strategies) can be effectively delivered in a mobile format [94]. Thus, the main objective is to assess the impact of the app on homework completion compared with CBT treatment-as-usual (ie, paper-based homework delivery). We will also obtain end user evaluations of the app’s usability in the context of treatment. Finally, we will also conduct exploratory analyses to assess the impact of the app on symptom improvement and cost-effectiveness, and to examine associations between cognitive profiles and homework completion as well as user experience.

#### Study Design

We will conduct a multisite, randomized controlled pragmatic clinical trial in routine clinical settings to test the app’s effectiveness under real-world conditions in order to enhance external validity and ensure successful implementation without sacrificing scientific rigor. A pragmatic clinical trial also suits the nature of mHealth technologies, which require ongoing improvement and are subject to rapid technological change [95-97]. After pretreatment assessment, participants will be randomly assigned to receive a manualized CBT treatment either with app support (app group) or without it (no-app group). Youth assigned to CBT with the app will receive CBT treatment for 12 weeks with the app included (app group). They and their therapists will be shown how to use the app. Youth assigned to receive CBT treatment for 12 weeks with paper-based homework will be in an active control condition (no-app group). Both groups will be in treatment for 12 weeks, the typical duration of CBT. However, therapists in both the app and no-app groups will be able to provide additional sessions should they deem it clinically relevant. Duration of CBT will be recorded and group differences will be analyzed. Following the pretreatment assessment and exclusions, participants providing informed consent will be randomly assigned to treatment group. Random assignment will be done at each test site separately by the site coordinator. At each site, half the participants will be assigned to each group in order to control for site-specific variables (eg, type of CBT delivered, professional background of therapist). To assess the app’s effectiveness both immediately and over time, assessments will be done at the end of the 12-week treatment (posttreatment) and again after 6 months (follow-up 1) and 12 months (follow-up 2). At each time-point, participants will receive a CAD$30 gift card as an honorarium.

#### Sample Size and Power

Given the low rates of homework completion in research to date, we expect that the app will have at least a moderate effect on homework completion compared with the no-app group [28,35,36]. A power calculation, based on a comparison between 2 groups (app vs no-app) across 4 occasions (pre, post, follow-up 1, follow-up 2) for a single measure, indicates that a sample size of 35 for each group would provide power of 0.84 to detect a moderate effect size for homework completion at α = .05. To allow for dropouts [98], we will recruit 100 youth between 12 and 18 years of age (10 per group at each site). As noted earlier, treatment dropout rates can be substantial and adherence to homework is often quite low. This has been taken into account in calculating the sample size. The expectation is that compliance will be greater for the app than the no-app group. Based on previous research with youth [98], we expect a 10% rate of attrition at each of the 2 follow-ups.

#### Recruitment

Recruitment will be the same as in Phase 2. In previous work in our clinics, consent rates have been 80% or higher. Interest in using the app is expected to be high. Typically, intake at the test sites is approximately 1 per week and the wait-list period is at least 12 weeks for noncrisis patients. Therefore, based on intake and consent rates and assuming sequential recruitment, we will recruit at a rate of 5 youth per week across the 5 sites, reaching an overall sample of 100 within 6 months. Posttreatment assessments would be completed at the end of treatment (ie, 3 months later), and the follow-ups 6 and 12 months thereafter. The same inclusion and exclusion criteria apply as in Phase 2.

#### Measures

At pretreatment, as in Phase 2, we will assess (1) demographic characteristics and comfort with technology to characterize the sample, and (2) cognitive skills to examine associations between cognitive profiles and homework completion as well as user experience.

During treatment, homework completion will be assessed weekly. The quantity (amount) as well as the quality (appropriateness) of homework will be assessed, based on evidence that they both relate to treatment outcome [99]. Multiple sources of homework information (adolescent, therapist, data logged by the app) will be obtained to address potential differences between sources [99,100]. Adolescents will assess the quantity and quality of their homework upon arriving at the session, by rating on a 5-point scale how much of it was completed and how well it was done, using 2 items from the Homework Rating Scale II [101], an internally consistent measure of homework quantity and quality developed for use with adults [102]. The 2 items have not previously been tested with adolescents and will be pilot-tested in this study. Two additional measures of homework quantity will be obtained by asking adolescents to estimate the number of days and the number of hours they spent doing homework [103]. Therapists will assess homework completion following the session. They will assess homework quantity by rating the proportion of assigned homework that was completed (from 0% to 100%), a method used in previous research [36,103]. They will rate the quality of the work on a 6-point scale [104], using criteria specified for each assignment; this measure has been found to predict treatment outcome [103]. A third source of information about homework quantity will be obtained in the app group from data logged by the app: the proportion of the task completed, the time spent on the task, and the number of visits to the assignment page.

At pre, post, and each follow-up, treatment outcomes will be assessed. Anxiety and depression will be assessed using the same instruments as in Phase 2: the Achenbach Scales, which assess general symptomatology as reflected in internalizing problems, and externalizing problems, which often co-occur with anxiety and depression [105], the MASC-2, the CDI-2, and the ADIS. Other outcomes also will be assessed. Maladaptive cognitions will be assessed using the Children’s Automatic Thoughts Scale [106], in which youth rate the frequency over the past week of a set of 40 self-statements describing negative thoughts about physical threats, social threats, personal failure, and hostile intent. Quality of life will be assessed by the Pediatric Quality of Life Inventory [107,108], in which youth rate 23 self-statements assessing physical, emotional, social, and school functioning.

At posttreatment, a usability evaluation will be conducted with the app group (youth and therapists) following completion of the posttreatment assessment, using the same protocol as in Phase 2. To examine differences in client satisfaction between the app and no-app groups, will be assessed posttreatment (following the usability evaluation in the app group) via the Client Satisfaction Questionnaire (CSQ [109,110]), an 8-item global measure of satisfaction with service comprised of statements phrased as questions to be answered on a 4-point scale ranging from 1 (poor) to 4 (excellent). The CSQ is a well-established measure with good psychometric properties [110] that has been used in numerous studies with diverse patient samples, including children and adolescents in CBT [111,112]. Treatment fidelity will be assessed by an integrity-to-protocol checklist [113], which therapists will complete after each session. The checklist yields a proportion score reflecting the rate of adherence to the CBT treatment manual. Finally, health economic cost data will be obtained to assess cost-effectiveness of the app. Cost data will consist of technology costs, including detailed micro costing of the app program (eg, program costs, smartphone-related costs, server costs, maintenance), and the cost of time and other resources needed to integrate the app into routine clinical practice by therapists and clinics.

#### Data Analysis

To assess the app’s effectiveness, differences between the app and no-app groups will be examined with respect to homework completion. We will also explore group differences in treatment outcomes. Although randomization will be used to mitigate group differences, potential differences between groups will be examined. If differences are found on variables that may be related to homework completion or treatment outcome (symptom severity/diagnosis, age, sex distribution, history of psychotherapy and pharmacological interventions, comorbid mental health problems, therapist), these variables will be included as covariates in the analyses. Descriptive analyses will be performed on demographic characteristics, comfort with computers, and retention rates (participants assessed at posttreatment and follow-ups). To avoid bias in parameter estimates, missing data will be handled by performing intent-to-treat analyses, in which missing values are replaced using the last observation carried forward, a method appropriate for randomized designs [114].

##### Homework Completion

Effects of the app on homework completion will be analyzed separately for homework quantity and quality. Psychometric properties of the measures will be examined and correlations will be computed to examine congruence between adolescent and therapist sources of information. If warranted, aggregate scores will be computed; otherwise, separate analyses will be done using different measures. To test the hypothesis that the quantity and quality of homework will be greater in the app compared with the no-app group, we will test group differences in quantity and quality scores. We will also explore timing effects, given evidence suggesting that homework completion varies over time and may decrease over the course of treatment [36]. Scores for the first 4, middle 4, and final 3 weeks of homework will be averaged, creating 3 assessment points, and a mixed-model repeated measures multivariate analysis of the variance will be performed with the between factor of group (app vs no-app), the within factor of time (early, middle, late), and the 2-way interaction of group × time.

Exploratory analyses will also be performed. Subgroup analyses will explore differences in the app’s impact on homework completion as a function of test site and gender. Correlations will explore relations between cognitive profiles and homework completion. For the app group, psychiatric and cognitive assessment data will be aggregated and advanced analytics will be performed to examine predictive relations between patient attributes (anxiety, depression, cognitive profile) and the quantity and quality of homework completion. These analyses will include homework completion data logged by the app (proportion of the task completed, the time spent on the task, and the number of visits to the assignment page).

##### Treatment Outcomes

For exploratory purposes, we will examine group differences in treatment outcomes. Group differences in anxiety and depression will be analyzed for both dichotomous (diagnosis) and continuous (symptom severity) measures. For each diagnosis (presence vs absence), chi-square tests will be performed comparing the number of youth meeting criteria for diagnosis at pre versus post, pre versus follow-up 1, and pre versus follow-up 2. For each of the continuous measures of symptom severity, following the recommended approach for trials with pre, post, and follow-up measures [115], a repeated measures multivariate analysis of the covariant will be performed in which the between factor is group (app vs no-app) and the within factor is time (pre, post, follow-up 1, follow-up 2), to compare group and time effects and interactions between group and time, using the pretreatment value as a covariate. Where significant effects are found, simple contrasts will be conducted to ascertain where the significant differences lie. Group differences in maladaptive cognitions (overall score) and quality of life (overall score), will be analyzed in the same way. Effect sizes will be calculated comparing the effects of app vs no-app on each of the outcomes. Analyses will also be performed to establish clinical significance and the reliable change index [116]. Subgroup analyses will explore differences in the app’s impact on treatment outcomes as a function of test site and gender.

If the analyses reveal significant group differences in treatment outcomes, additional exploratory analyses will be performed to aid interpretation of the data. To explore whether better homework completion (greater quantity and/or quality) or something unique about the mobile technology is most likely responsible for group differences in treatment outcomes, we will examine associations between homework completion and treatment outcomes separately for the 2 groups. Mobile devices may increase patient engagement and empowerment [1,117] and reduce concern about the perceived stigma associated with receiving treatment [118], resulting in beneficial effects on treatment that are independent of homework completion. Accordingly, regression analyses (ordinary least squares, logistic) would be performed to predict each of the dichotomous (diagnosis) and continuous (symptom severity) outcomes at posttreatment, follow-up 1, and follow-up 2 from measures of homework completion, controlling for the pretreatment value of the outcomes. A finding of group differences in the strength of associations between homework completion and treatment outcomes would suggest that an improvement in homework completion is not the only mediator of app effects on treatment outcome.

Finally, if treatment outcomes are better for the app compared with the no-app group, exploratory mediation analyses will be performed for the app group to assess whether homework completion (quantity, quality) affects treatment outcomes indirectly through support for learning (ratings of how much the app supports access to assignments, memory and understanding of lessons, motivation to practice, coaching and suggestions, self-monitoring of symptoms and changes, review of homework). To test for mediation, we will perform tests of indirect effects using a regression-based path-analytic approach [119,120].

##### Usability Evaluation

To identify usability issues, content analysis will be performed on the usability evaluation data, as in previous phases. An exploratory qualitative analysis will be performed to examine associations between cognitive profiles and the usability evaluation data in order to further improve the user experience and customize it for different cognitive profiles.

##### Client Satisfaction With Service

To examine differences between the app versus no-app groups in client satisfaction, a *t*-test will be performed on total CSQ scores. Levels of satisfaction will be compared with norms established in other studies of youth in CBT treatment [111,121].

##### Treatment Fidelity

Proportion scores computed from the integrity-to-protocol checklist will be analyzed to compare the app and no-app groups with respect to therapists’ adherence to the CBT treatment manual.

##### Cost-Effectiveness of CBT with App Support

Cost-effectiveness will be computed for several treatment outcomes (anxiety, depression, quality of life) using the cost data described above (dollar values). An incremental cost-effectiveness ratio will be calculated for the measures at posttreatment and follow-ups 1 and 2. The analysis will use a health care sector perspective. Costs that are equal in both treatment alternatives will be excluded, because they would not impact the results. The study will use Ontario provincial list costs for health care services and market values for other resources when available. All costs will be shown in current values using the Canadian Consumer Price Index. Costs over 1 year will be discounted at a 5% discount rate and a sensitivity analysis will use 0% and 3% discount rates. We will follow Canadian Agency for Drugs and Technologies (2006) guidelines [122], the Institute of Health Economics economic evaluation report [123], and methods for costing the alternatives and performing the cost-effectiveness analysis (CEA) [124]. The cost and effectiveness outcomes will be further analyzed using economic decision modeling techniques. The modeling will include consideration of the uncertainty in both effectiveness measures and different cost variables using probabilistic sensitivity analysis techniques that allow simulation of the expected outcomes using a cost-effectiveness plane that shows the estimated incremental cost and effectiveness estimates and their mean value. Further, the results will be shown using the Cost Effectiveness Acceptability Curves, the most widely used tool to show the probability that the new technology will be accepted with different societal willingness to pay for it. The modeling part of the CEA will use state of the art modeling practices and International Society for Pharmacoeconomics and Outcomes Research good modeling guidelines [123,125,126].

## Results

Phase 3 will be completed by September 2019. Ethics approval has been received for Phase 1 of the study from the Research Ethics Board at the Centre for Addiction and Mental Health.

## Discussion

The app will be a unique adjunct to treatment for adolescents in CBT, focusing on both anxiety and depression, developed in partnership with end users at every stage from design to implementation, customized for different cognitive profiles, incorporating data analytics to support ongoing analysis and improvement, and designed with public domain depression symptom tracking measures for youth made interoperable with EMRs.

## Abbreviations

ADIS: anxiety disorders interview schedule
API: application programming interface
CBT: cognitive behavior therapy
CDI-2: Child Depression Inventory 2nd ed
CEA: cost-effectiveness analysis
CSQ: client satisfaction questionnaire
EMR: electronic medical record
FHIR: fast healthcare interoperability resources
IT: information technology
MASC-2: Multidimensional Anxiety Scale for Children 2nd ed
RA: research assistant
SMART: substitutable medical applications and reusable technology
SmartCAT: smartphone-enhanced child anxiety treatment

## Appendix

Multimedia Appendix 1 Peer Review Comments from CIHR.
