# Supplementary material for: Cognitive Behavior Therapy for Anxious and Depressed Youth: Improving Homework Adherence Through Mobile Technology
Source: JMIR Res Protoc. 2016 Nov 10;5(4):e209. doi: 10.2196/resprot.5841 (PMC5122719; doi:10.2196/resprot.5841)
Supplement: Multimedia Appendix 1 [file resprot_v5i4e209_app1.pdf]

**Review Type/Type d'évaluation:**	SO Notes /Notes de l'agent scientifique
**Name of Applicant/Nom du chercheur:**	Wilansky, Pamela
**Application No./Numéro de demande:**	348308
**Agency/Agence:**	CIHR/IRSC
**Competition/Concours:**	2015-06-03 Operating Grant: eHealth Innovations Initiative: eHealth Innovation Partnership Program (eHIPP)/Subvention de fonctionnement: Initiative Innovations en cybersanté : Programme de partenariats pour l'innovation en cybersanté (PPIC)
**Committee/Comité:**	eHealth Innovation Partnership Program (eHIPP): Youth and Adolescents Mental Health/Programme de partenariats pour l'innovation en cybersanté-Des jeunes et la santé mentale
**Title/Titre:**	Cognitive Behavior Therapy for Anxious and Depressed Youth: Improving Outcomes through Mobile Technology

Assessment/Évaluation:

This application proposes developing and testing a cognitive behavioral therapy (CBT) app for anxiety and mood disorders. The process flows and connection to clinical needs was well thought through and supported. Other strengths include the youth involvement in the project , including the design of the app and having formal roles. Another strength is that naming of the app is not stigmatizing to end users. The committee was confident that the team and the project are feasible to implement. Outcome measures related to use of the app by end users were unclear. The extent of innovation introduced by the app in the area of CBT treatment was uncertain, as it is an add-on to traditional 12 week intensive care treatment.
